# Cisplatin-Induced Bradycardia: A Silent Risk Observed in Two Different Clinical Cases

**DOI:** 10.7759/cureus.19769

**Published:** 2021-11-20

**Authors:** Sameen Bin Naeem, Musa Azhar, Naqib Ullah Baloch, Mansoor Abbas, Muhammad Waheed, Rizwan Masood Sheikh

**Affiliations:** 1 Medical Oncology, Shaukat Khanum Memorial Cancer Hospital and Research Centre, Lahore, PAK

**Keywords:** cisplatin-induced cardiotoxicity, cardiotoxicity, germ cell tumour, bradycardia, cisplatin

## Abstract

Cisplatin is a platinum-containing drug that inhibits DNA synthesis by inhibiting cross-linking, denaturing DNA strands. It is used in combination with other chemotherapeutic agents to treat several types of cancers. Numerous adverse effects have been reported with this compound. However, it is considered a safe medication in terms of cardiotoxicity. In this report, we discuss the case of two patients who experienced bradycardia while receiving cisplatin as part of combination therapy. A workup was undertaken to rule out other possible causes of bradycardia, and the diagnosis of cisplatin-induced bradycardia was made.

## Introduction

Cisplatin was first described by Michele Peyrone in 1845 [1], and it was approved for use in ovarian and testicular cancers in 1978. It is a DNA synthesis inhibitor and has a half-life of 24 hours to 47 days. Depending on the chemotherapy protocol, it is administered intravenously alone or in combination with other medicines, according to the protocol described in the regimen. The most commonly reported side effects are nausea, vomiting, nephrotoxicity, ototoxicity, myelosuppression, anaphylaxis, and alopecia. It is usually considered a cardio-safe drug with no monitoring required for cardiotoxicity [2,3].

We present the case of two patients who developed bradycardia while receiving cisplatin as part of combination therapy. An extensive workup was performed to rule out other probable causes of bradycardia, which led to the diagnosis of cisplatin-induced bradycardia.

## Case presentation

Case 1

A 37-year-old man with no pre-existing conditions presented with left testicular swelling for three months. He was diagnosed with testicular germ cell tumor stage II-B. He was offered combination therapy with cisplatin, etoposide, and bleomycin. His baseline investigations, including complete blood count, liver, and renal functions, were within normal limits before initiating chemotherapy. On the second day of chemotherapy, after finishing cisplatin, the patient started having chest heaviness and palpitations.

**Figure 1:**
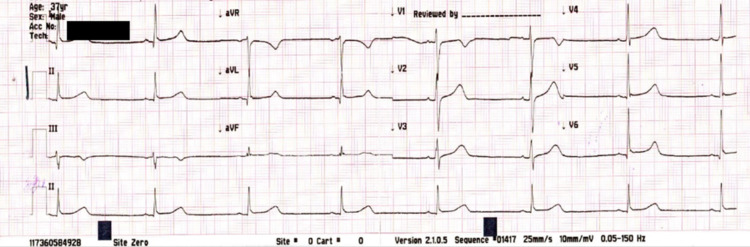
ECG showing bradycardia (heart rate of 43/minute) ECG: electrocardiogram

Further examination revealed a heart rate of 58 beats/minute, which had dropped from a baseline heart rate of 100 beats/minute. Upon observation, his heart rate further dropped to 43 beats/minute over the span of eight hours. ECG revealed no evidence of ischemia or arrhythmia. However, the QTc interval was 471 ms, which was prolonged as per the Bazett formula (Figure 1). He was kept under monitored observation for any further drop in heart rate. Myocardial ischemia was ruled out with serial ECGs and cardiac enzymes. Detailed workup for bradycardia eventually returned negative, including serum electrolytes level and thyroid workup (Table 1). There was no previous history of rate-limiting medicines, which led us to the diagnosis of cisplatin-induced bradycardia. He was kept in a high dependency unit for cardiac monitoring, where the cardiologist was also reviewing him daily. His bradycardia improved after 48 hours of initial presentation, which was confirmed on serial ECGs. He was discharged home after cardiology consultation on follow-up. The stress echo performed at another facility was reported to be normal.

He was followed up two weeks later in the OPD, and he had remained stable in the interim with no further episodes of chest heaviness or apprehensions.

The patient has completed his remaining three cycles of combination chemotherapy with carboplatin, etoposide, and bleomycin. His scans and tumor markers are stable and he is currently referred to surgical oncology for retroperitoneal lymph nodes dissection.

Case 2

A 32-year-old male was diagnosed with T cell-rich B-cell lymphoma, stage IV A, who initially had a complete metabolic response after six cycles of R-CHOP [rituximab, cyclophosphamide, doxorubicin hydrochloride (hydroxydaunorubicin), vincristine sulfate, and prednisone] with three cycles of intrathecal methotrexate. When he relapsed with biopsy-proven extensive marrow involvement, R-DHAP (rituximab, cytarabine, cisplatin, and dexamethasone) was commenced as second-line therapy.

All his baseline investigations were within normal limits before commencing chemotherapy. On receiving cisplatin 100 mg/m^2^ on day one, the patient started having chest discomfort with no complaints of shortness of breath or palpitations.

**Figure 2:**
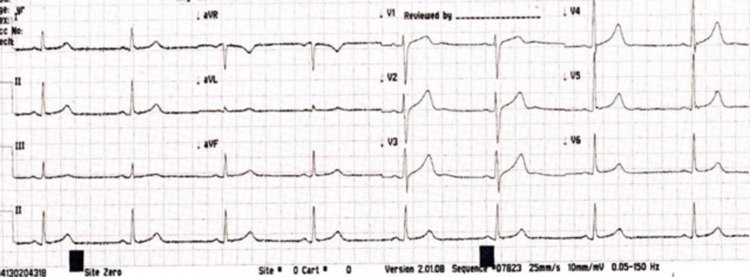
ECG showing heart rate of 45/minute during cisplatin infusion ECG: electrocardiogram

Further examination revealed a heart rate of 46 beats/minute with a baseline heart rate of 76 beats/minute. ECG did not show ischemia or arrhythmia, with QTc of 455 msec as per the Bazett formula, as shown in Figure 2. He recovered uneventfully within the next four hours, requiring no immediate intervention to stabilize his heart rate except stopping the chemotherapy. Workup for bradycardia was negative, including markers for cardiac ischemia, electrolytes imbalance, and thyroid profile (Table 1); there was no previous history of rate-limiting medications. The cardiologist advised continuing cardiac monitoring and outpatient stress echocardiography, which was later reported to be normal. He was kept under monitored observation afterward and received the remaining chemotherapeutic agents as per schedule with no adverse events.

Cisplatin was substituted with carboplatin; the patient completed his remaining three cycles, and his heart rate remained stable during carboplatin infusions. He was regularly seen by a cardiologist till the completion of chemotherapy, but no further episode of bradycardia was observed. An end-of-treatment scan indicated new uptake in the marrow surrounding the left acetabulum and first left costochondral junction. The patient then received salvage combination chemotherapy including rituximab, etoposide, carboplatin, and ifosfamide with the end-of-treatment scan suggesting complete metabolic response. Currently, the patient is on the autologous stem cell transplant list; his stem cells have been harvested.

**Table 1:** Lab parameters of both cases TSH: thyroid-stimulating hormone

Parameter	Normal values	Case 1	Case 2
Calcium, corrected (mg/dL)	8.5–10.5	8.91	9.24
Serum magnesium (mg/dL)	1.6–2.6	2.84	2.21
Serum potassium (mmol/L)	3.5–5.1	4.85	4.23
Serum sodium (mmol/L)	136–145	140	133
Serum phosphorus (mg/dL)	2.5–4.5	4.52	3.11
Serum TSH (mIU/L)	0.4–4.0	2.6	3.4
Serum creatinine (mg/dL)	0.70–1.20	0.73	0.54
Troponin I (ng/mL)			
1st	less than 0.046	0.005	0.003
2nd		0.003	<0.003
3rd		0.006	<0.003

## Discussion

Cisplatin is a platinum compound that interferes with DNA replication; it is usually given in combination therapy and is a mainstay in treating many types of cancers. This drug is highly effective against testicular cancer when combined with bleomycin and etoposide, with a survival rate of 90% in suitable-prognosis patients, and 75% and 50% in intermediate- and poor-prognosis patients respectively [4]. It has also shown efficacy in the treatment of other tumors such as ovarian, lung, head and neck, and bladder cancers, as well as sarcomas and lymphomas [3]. Combination therapies with other chemotherapeutic agents have been used to overcome resistance and potential side effects of cisplatin. Nausea and vomiting are the most commonly reported side effects of cisplatin-based therapies. Other notable side effects include ototoxicity, nephrotoxicity myelosuppression, and anaphylaxis [5]. It is usually considered safe from the cardiac point of view. However, there have been a few cases reports with cardiotoxicity, hypotension, or reduced ejection fraction [6-9]. Buza et al. have also reported that there are only case reports of sinus bradycardia with cisplatin in the literature [10]. The present data, although scarce, suggests screening all the patients for any heart-related illnesses and any rate-limiting drugs before commencing cisplatin-based regimens to minimize the risk of platinum-induced cardiotoxicity [11,12]. Patients receiving such regimens should be monitored closely for left ventricular ejection fraction, and ECG monitoring should be employed for early detection to reduce drug-related complications and for better outcomes [6,13]. Patients should be advised to maintain a healthy lifestyle [14].

Cisplatin-based chemotherapy regimens have been associated with cardiac ischemia, hypertension, and diastolic dysfunction [15], among other side effects. There is enough clinical evidence to indicate that cisplatin may have exerted a direct toxic effect on cardiac muscles in such cases, as evidenced by the observation of bradycardia occurring exclusively during infusions and the temporal relationship between cisplatin administration and the development of bradycardia followed by normalization of heart rate upon discontinuation [11]. Some authors have suggested that cisplatin accumulates in the sino-atrial node, causing cardiotoxicity expressing as atrioventricular nodal blocks [16]. It is difficult to ascertain the exact cause of bradycardia in our case; however, the immediate disappearance of bradycardia upon discontinuation and absence of nodal blocks on ECG suggest that it could be due to the direct effect of the drug on the cardiac muscle.

## Conclusions

Cisplatin-induced bradycardia, which is scarcely reported in the literature, should be taken seriously. There are only case reports of cisplatin-induced bradycardia in the litrature. Our report has consolidated the existing data, which can contribute to defining guidelines for workup before offering cisplatin to patients. Oncologists should keep in mind this rare but important side effect of this wonder drug, which has significantly improved outcomes in cancer therapeutics. Patients who are currently on rate-limiting drugs, such as beta-blockers and calcium channel blockers, should be monitored closely, as these patient subgroups are predisposed to developing bradycardia.
